# A Phase I Trial of Allogeneic γδ T Lymphocytes From Haploidentical Donors in Patients With Refractory or Relapsed Acute Myeloid Leukemia

**DOI:** 10.1016/j.clml.2023.02.003

**Published:** 2023-05

**Authors:** Jan Vydra, Emilio Cosimo, Petr Lesný, Richard Sebastian Wanless, John Anderson, Alan George Clark, Angela Scott, Emma Kate Nicholson, Michael Leek

**Affiliations:** 1 Institute of Haematology and Blood Transfusion, Prague, Czech Republic; 2 TC BioPharm, Motherwell, UK; 3 UCL Institute of Child Health, London, UK; 4 The Royal Marsden Hospital, London, UK

**Keywords:** γδ T cells, Adoptive immunotherapy, Acute myeloid leukemia, Haploidentical donors, Phase I trial

## Abstract

**Introduction** We report the results of a phase I clinical trial NCT03790072 of an adoptive transfer of γδ T lymphocytes from haploidentical donors in patients with refractory/relapsed acute myeloid leukemia after lymphodepletion regimen. **Patients and methods** Healthy donor mononuclear cells collected by leukapheresis were consistently expanded to generate products of 10^9^ to 10^10^ γδ T cells. Seven patients received donor-derived T cell product at doses of 10^6^/kg (n = 3), 10^7^/kg (n = 3), and 10^8^/kg (n = 1). **Results** Four patients had bone marrow evaluation at day 28. One patient had a complete remission, one was classified as morphologic leukemia-free state, one had stable disease and one had no evidence of response. In one patient, there was evidence of disease control with repeat infusions up to 100 days after first dosing. There were no treatment-related serious adverse events or treatment-related Common Terminology Criteria for Adverse Events grade 3 or greater toxicities at any dose level. Allogeneic Vγ9Vδ2 T cell infusion was shown to be safe and feasible up to a cell dose of 10^8^/kg. **Discussion** In agreement with previously published studies, the infusion of allogeneic Vγ9Vδ2 cells was safe. The contribution of lymphodepleting chemotherapy to responses seen cannot be ruled out. Main limitation of the study is the low number of patients and interruption due to COVID-19 pandemic. **Conclusion** These positive Phase 1 results support progression to phase II clinical trials.

## Microabstract

Blood from healthy donors was consistently expanded to generate products of 10^9^ to 10^10^ γδ T cells. γδ T cell infusion in 7 patients was safe and feasible up to a cell dose of 10^8^/kg and with preliminary evidence of disease activity in acute myeloid leukemia.

## Introduction

Clinical studies have been reported in which autologous γδ T lymphocytes have been generated and infused in cancer patients.^1^, ^2^, ^3^ All these studies have employed predominantly a Vγ9Vδ2 subset through the use of bisphosphonate for expansion, and all have evaluated relatively small numbers of patients with solid or hematologic malignancies to identify feasibility and safety data. Our own earlier, open-label clinical trial evaluated the tolerability and feasibility of infusions of autologous peripheral blood-derived, *ex vivo* expanded γδ T cells. The study concluded that the generation of autologous γδ Τ cell products is feasible and safe for solid tumor patients up to a dose of 9 × 10^9^ total cells.^4^ However, the trial recruited only 8 patients over 2.5 years with multiple manufacturing failures in other patients.

Acute myeloid leukemia (AML) is a blood cancer affecting adults and children, characterized by infiltration of bone marrow and other tissues by clonally proliferative immature myeloid cells. While AML may be cured using chemotherapy, allogeneic stem cell transplantation and novel targeted therapies in select cases, significant proportion of patients have either relapsing or refractory disease. New treatment approaches are therefore a research priority.^5^ Appropriately stimulated Vγ9Vδ2 T cells have potent activity against AML blasts.^6^^,^^7^ This prompted the initiation of a study to evaluate the feasibility of adoptive transfer of allogeneic γδ T cells combined with lymphodepletion.

This article describes a phase I clinical trial of an allogeneic γδ T cell product of the Vγ9Vδ2 subtype, expanded using zoledronic acid and interleukin-2 (IL-2) in patients with relapsed or refractory AML.

## Materials and Methods

The study was designed as an open-label, safety and efficacy, escalating dose, single arm study on 9 adult patients (3 cohorts) and 3 + 3 design was to be used. Human leukocyte antigens (HLA) typed patients and potential blood-related donors were screened for comorbidities. Suitably matched or haploidentical family donors were selected according to protocol specified criteria and institutional guidelines of participating site. There was no formal sample size calculation for the study; a standard 3 + 3 dose escalation design was to be employed.

Adult patients with relapsed or refractory AML with greater than 5% of blasts in bone marrow or peripheral blood with a life expectancy of at least 3 months and Karnofsky performance score >50% were included in the study. Patients needed to be unsuitable for high dose salvage chemotherapy and/or allogeneic hematopoietic stem cell transplant and have a related HLA-haploidentical or HLA-matched donor. Full eligibility and exclusion criteria of patient and donor are shown in Supplemental Tables 1A and B.

Mononuclear cells were collected by apheresis from healthy donors. The apheresis starting material was then shipped to TC BioPharm (TCB) to undergo processing, with all remaining aspects of the manufacturing process carried out internally at TCB's Manufacturing Facility. Peripheral blood mononuclear cells (PBMCs) were isolated from the apheresis starting material via density gradient centrifugation, using semiautomated devices to minimize open vessel manipulations and variability. The PBMCs were then resuspended in cryoprotectant and aliquoted into vials at the required concentrations to create intermediate cell aliquots (ICAs). ICAs were submitted to the quality control department for controlled rate freezing and storage in vapor phase liquid nitrogen (vLN_2_). The cryobank then underwent relevant release testing including sterility testing, cell counting, viability and immunophenotyping testing. To create a batch, a suitable number of tested ICAs were released from the cell bank and transferred into the Manufacturing Facility for processing. The PMBCs were controlled rate thawed, resuspended, and initiated into closed system flask(s) with proprietary media, along with supplements to induce the selective expansion of the gamma delta (γδ) T cells. The cells then underwent a 14-day expansion process with a number of refeeds with fresh media supplemented with a xeno-free alternative to human plasma. After expansion, the cells were washed and concentrated before labeling the T cell receptor α/β positive (TCRα/β+) cells with TCRα/β-Biotin. Anti-Biotin Reagent was used for the magnetic depletion of TCRα/β+ cells from the cellular product. Depleted product was then resuspended at the required concentration and formulated in sodium chloride solution for infusion.

Patients were administered lymphodepleting chemotherapy with fludarabine 25 mg/m^2^ (max. dose 50 mg) from day −6 until day −2 and cyclophosphamide 500 mg/m^2^ on days −6 and −5. Haploidentical γδ T lymphocyte product was administered on day 0.

**Figure 1:**
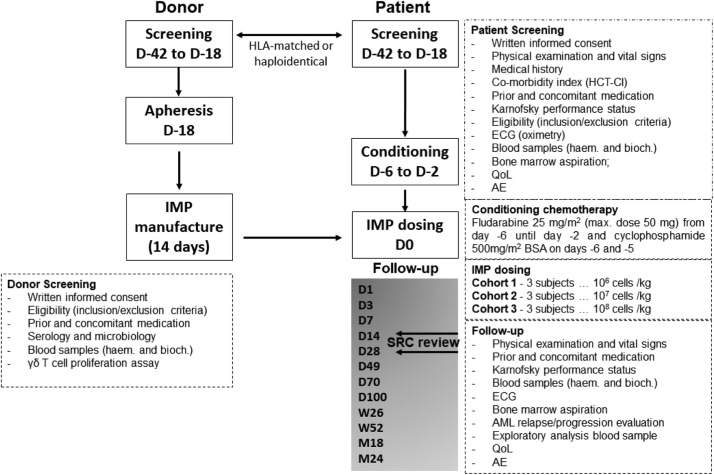
TCB-202-001 Trial schematic. D denotes day. Donor and patient screening commenced between day –42 and –18. Apheresis of donor material was completed by day –18. OmnImmune (the Investigational Medicinal Product - IMP) manufactured within 14 days. Patients received conditioning chemotherapy from day –6 to –2. IMP dosing on day 0. Follow-up from day 1 through 2 years. SRC denotes safety review committee. SRC assessed safety and efficacy on day 14 and day 28. The trial was conducted in accordance with the ethical principles of the Declaration of Helsinki and the International Council of Harmonization Guidelines on Good Clinical Practice. All patients provided written informed consent.

The schedule of investigation assessed disease burden and response, detected infused products, and monitored toxicity (Figure 1). Primary endpoints were assessment of adverse events and dose limiting toxicities for 28 days following infusion, and establishment of maximum tolerated dose. Secondary endpoints were complete remission rate, overall response rate and quality of life assessments. Dose escalations of 1, 10, and 100×10^6^ cells/kg with 3 patients per dose cohort were dependent on dose limiting toxicity (DLT).

The statistical analysis was descriptive in nature. The incidence of DLTs was summarized descriptively by γδ T cells dose for evaluable subjects. In this case, evaluable patients consisted of all patients completing the period of evaluation for DLTs or who experienced a DLT before completing that period. Safety data from each cohort were reviewed by the safety review committee (SRC) after the completion of the DLT evaluation period for each cohort. Since no statistical testing was performed, no adjustment for interim analysis was required. All other data will be summarized descriptively by γδ T cells dose for all treated patients.

### Flow Cytometry

Flow cytometry analysis of exploratory samples was conducted in a non-GCLP research laboratory at TCB, for research purposes only. Fluorophore-labelled antibodies for HLA-A2, HLA-B7, CD3, TCR Vγ9, CD45, CD33, CD14 and CD16 were purchased from Biolegend (San Diego, CA). Fluorophore-labelled antibodies for HLA-A3 and TCR α/β were purchased from Miltenyi Biotec (Bergisch Gladbach, Germany). Antibody staining was performed according to the relevant manufacturers’ instructions. Measurements were done on a NovoCyte flow cytometer (Agilent, Santa Clara, CA). Data were analyzed with NovoExpress software (Agilent, Santa Clara, CA). For analysis of patient trial samples, PBMCs were cryopreserved at the clinical site and were subsequently shipped to TCB for assessment of detection of product-specific HLA-type, indicating presence of donor cells. The proportion of CD3+ / Vγ9+ cells in PBMCs samples from patient PRA1-5006 were analyzed for the expression of HLA-B7, HLA-A2, and HLA-A3 over time, up to 100 days post infusion. Donor-specific cells were identified based on differential expression of HLA markers between donor and recipient over time, up to 100 days post infusion.

## Data Availability Statement

The data were generated by the authors and are publicly available at Open Science Framework.

## Results

**Figure 2:**
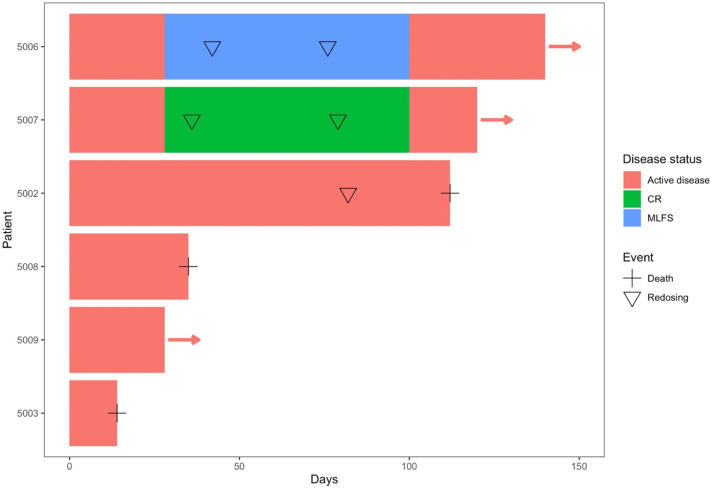
Survival and disease status from infusion in evaluable patients.

The TCB-202-001 trial opened in November 2018 and was terminated prematurely in March 2020 due to the COVID-19 pandemic. Ten patients were recruited of which 2 patients (5004, 5005) failed screening and 2 could not be followed up due to early trial closure: of these, 1 (5010) did not have day 28 marrow evaluation, whilst 1 patient (5011) did not receive a planned infusion. A patient summary is depicted in Table 1 and Figure 2. Details of the HLA status of donors and patients are in Supplemental Table 2.

**Table 1:** TCB-202-001 Patient Summary

Patient	Age	Gender	AML Diagnosis	AML Genetics	Previous Therapy^a^	Initial TCB Infusion Dose	Baseline Bone Marrow Blast Count	D 14 Bone Marrow Blast Count	D 28 Bone Marrow Blast Count	Baseline Peripheral Blood Blast Count	D 14 Peripheral Blood Blast Count	D 28 Peripheral Blood Blast Count	Best Response^b^	Outcome
5002	65	F	AML – refractory relapse	Normal karyotype	- “3+7“, 2xIDAC, ALLO-HCT - LD-AraC + DLI - “3+7“+ DLI	(1 × 10^6^/kg)	62.8%	28.5%	5%	0%	3.3%	4.4%	SD	Redosing d 82 with initial response followed by disease progression and death on d 112
5003	49	M	AML – refractory relapse	Complex karyotype, TP53 mut.	- “3+7“, FLA/DAU, ALLO-HCT - FLA/IDA	(1 × 10^6^/kg)	38.8%	N/A	N/A	16.3%	N/A	N/A	N/A	Bilateral pneumonia from d 7 leading to death on d 14, not considered IMP related
5004	Screening failure													< 5% bone marrow blast level
5005	Screening failure													Screening failure due to refractory thrombocytopenia
5006	62	F	Secondary AML (breast cancer) refractory relapse	t (8;21)	- “3+7“, HIDAC - LD-AraC	(1 × 10^6^/kg)	51.2%	8.4%	2.6%	24.6%	0%	10.7%	MLFS	Re-dosing d 42 and 76. Disease progression d 100 followed by other novel treatment
5007	66	F	Relapsed AML	+8, *DNMT3A* mutation	- “3+7“, 3xIDAC, Atezolizumab +BL8040 maintenance	(1 × 10^7^/kg)	9.0%	4.6%	3.6%	2.3%	0%	0%	CR	Re-dosing d 36 and 79, remained in remission through d 100 at which time BM aspirate showed 5.2% blast count. Thereafter relapse and alternative treatment given
5008	48	F	Secondary AML (MPD), primary refractory	*CALR* mutation	- Alo-HCT - “3+7“ - FLA/IDA	(1 × 10^7^/kg)	14.8%	N/A	N/A	28%	N/A	N/A	N/A	Patient did not meet entry criteria (active infection) but treated on ethical grounds. Discontinued d 14 due to ongoing AML
5009	69	F	AML, refractory relapse	inv(16)	- “3+7“, 2xIDAC, LD-AraC - Venetoclax	(1 × 10^7^/kg)	66.6%	38%	75%	9.7%	4.8%	60%	NR	Study discontinued due to COVID
5010	59	M	Secondary AML (MPD), primary refractory	Normal karyotype	- “3+7”, FLAIDA, azacytidine	(1 × 10^8^/kg)	20.0%	N/A	N/A	17.2%	16.5%	N/A	NR	Study discontinued due to COVID
5011	68	F	AML	Complex karyotype	Azacitadine, LD-AraC	N/A	36.0%	N/A	N/A	56.2%		N/A	N/A	Study discontinued due to COVID

Abbreviations: ALLO-HCT = allogeneic hematopoietic cell transplantation; CR = complete response; FLA/DAU = fludarabine, cytarabine, daunorubicin; FLA/IDA = fludarabine, cytarabine, idarubicin; HIDAC = high-dose cytarabine; IDAC = intermediate-dose cytarabine; LD-AraC = low-dose cytarabine; MLFS = morphologic leukemia-free state; NR = no response; SD = stable disease.

a Previous therapy = each bullet point corresponds to 1 chemotherapy treatment regimen or stem cell transplant: “3+7”: standard dose cytarabine with daunorubicin or idarubicin.

b Responses were evaluated according to ELN 2017 criteria.^14^

Seven patients (5002; 5003; 5006; 5007; 5008; 5009, and 5010) received donor-derived T cell products all with >5% baseline blasts in bone marrow. Five survived to day 14 post-treatment. Patient 5003 died from infection on Day 14, categorized by the SRC as unlikely to be drug-related, while patient 5008 discontinued on day 14 due to disease progression. Four patients (5002; 5005; 5007, and 5009) had 28-day bone marrow evaluation, when 1 (5007) had complete remission (CR), 1 (5006) was classified morphologic leukemia-free state (MLFS), 1 (5002) had stable disease (SD) and 1 (5009) had no evidence of response. Weekly toxicity evaluations were performed through day 28 to determine dose-limiting toxicities.

Treatment emergent adverse events classified as serious or treatment-related are summarized in Table 2. Complete listing of adverse events by common terminology criteria for adverse events (CTCAE) term and grade is provided in Supplemental Table 3A and 3B. No graft versus host disease or neurotoxicity was observed. One patient experienced a possible cytokine release syndrome grade 1, although the symptoms were not specific. There were no treatment-related serious adverse events or treatment-related CTCAE grade 3 or greater toxicities at any dose level.

**Table 2:** TCB-202-001 Summary of Treatment Emergent AEs

	Number of Events	Dose of CELLS
Any TEAE	Total: 73	
	55	1 × 10^6^
	11	1 × 10^7^
	7	1 × 10^8^
Related TEAE^a^	Total:2	
	2	1 × 10^6^
Serious/severe TEAE^b^	Total:4	
	3	1 × 10^6^
	1	1 × 10^7^

TEAE = treatment emergent adverse event.

a Two patients were reported to have related TEAE. These events were pyrexia of CTCAE grade 1 in 1 patient and increased C-reactive protein of CTCAE grade 2 in 1 patient. These events were not classified as serious adverse events.

b In total 4 patients experienced a SAE. These were febrile neutropenia of CTCAE grade 3 in 1 patient, *Clostridium difficile* colitis of CTCAE grade 3 in 1 patient, gastrointestinal hemorrhage of CTCAE grade 4 in 1 patient and pneumonia of CTCAE grade 5 leading to death in 1 patient. These SAE were not considered to be related to study medication.

### Persistence and Activity of Allogeneic Vγ9Vδ2 T Cells Following Adoptive Transfer

**Figure 3:**
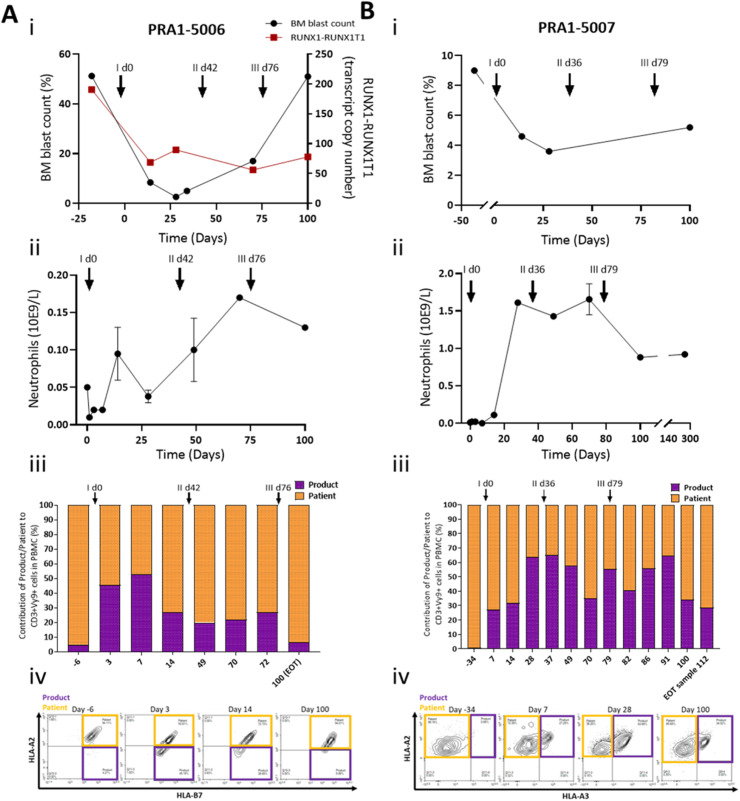
Clinical response and Vg9d2 T cell persistence in blood in patients PRA1-5006 (A) and PRA1-5007 (B). Bone marrow (BM) blast % (i) and neutrophil counts (ii) were performed at the clinical site at various time points. Graphs presented in i and ii are extrapolations from clinical data generated at the clinical site. For patient PRA1-5006, MRD (dark red symbols, A, i) was monitored by measuring RUNX1-RUNX1T1 transcript levels arising from t (8;21) by real-time qPCR. Proportional contribution (%) of Product/Donor to Vγ9δ2 T cell (iii) in patient blood samples (taken at the clinical site) was assessed by flow cytometry at TC BioPharm for research purposes only, utilizing the partial Product/Patient HLA mismatch (iv). Black arrows with i, ii, and iii indicate time of first, second, and third dose, respectively.

Patients surviving beyond 28 days were re-dosed without further lymphodepletion. In 3 patients who had CR (5007), MLFS or SD (5002, 5006) at the day 28 assessment, the protocol criteria were met for redosing with dosing based on the advice of the trial steering committee. No dose limiting toxicities were documented following repeat infusion. In patient 5002, initial infusion with the lowest dose of 1 × 10^6^/kg resulted in MLFS status at day 28 and a second infusion at the higher dose of 1 × 10^7^/kg were administered on day 82 resulting in a transient normalization in full blood count. Patients 5006 and 5007 received 2 further infusions at the same dose as the initial infusion (1 × 10^7^/kg). Patient 5006 was the only subject in the study to have had serial bone marrow samples and an appropriate measurable residual disease (MRD) marker available for correlation with bone marrow blast count. In this patient, MRD evaluations using the AML1-ETO fusion product by real-time qPCR^8^ and bone marrow aspirates were performed on days 28 and 34 and at time points following the second and third infusions. In this patient, the MRD levels showed a sustained reduction from the baseline value (Figure 3). However, there was subsequent increase in bone marrow blast count after the second and third infusions indicative of disease progression. In patient 5007 who had achieved CR on day 28, further infusions were administered on days 36 and 79. The patient's full blood count was normalized between days 28 and 70 consistent with ongoing complete remission. At day 100 the bone marrow blast count remained suppressed at 5.2% (Figure 3B). HLA typing of patients 5006 and 5007 and their donors was performed to identify mismatches to distinguish infused donor derived from host γδ T cells in serial blood samples (Supplemental Table 2). In both patients there was sustained detection of adoptively transferred donor-derived T cells in blood samples for at least 100 days following the initial infusion (Figure 3). Because of the repeat infusions given to patients 5006 and 5007, it is not possible to evaluate the long-term capacity for engraftment.

## Discussion

This article describes the feasibility, safety, and tolerability of an allogeneic haploidentical γδ T lymphocyte product, which can become an off-the-shelf therapeutic, suitable for investigation of therapeutic potential across multiple indications. Strategies to elicit durable clinical response are required for the treatment of myeloid malignancies.

γδ T lymphocytes represent an emerging field with several previous clinical trials in small numbers of patients providing preliminary evidence of safety and feasibility. A 2014 systematic review of clinical trials involving in vivo expansion or adoptive transfer of γδ T lymphocytes identified 12 relevant studies including 157 patients, of which 7 trials (77 patients) involved adoptive transfer of *ex vivo* expanded γδ T cells. Objective responses were consistent with efficacy superior to standard of care, second-line therapy for renal and prostate cancer, but not for non-small cell lung cancer.^2^ A more recent review^3^ identified another 5 studies^9^, ^10^, ^11^, ^12^, ^13^ involving 98 patients with adoptive transfer of autologous γδ T cells. Pooled data from these 5 recent trials give objective response rate of 26%. Thus, the literature supports potential anticancer activity of autologous γδ T cells following adoptive transfer.

Recruitment to an earlier clinical study of autologous *ex vivo* expanded gamma delta T cells demonstrated a high incidence of poor cell expansion and manufacturing failure, arguing in favor of an allogeneic approach. A pilot study in patients with advanced and refractory blood cancers who were not eligible for hematopoietic cell transplantation (HCT) due to lack of disease control^14^ showed that the adoptive cell transfer (ACT) of γδ T cells from an allogeneic-related haplo-identical donor source is both feasible and safe. The MHC independence of γδ T cells also supports their use as an allogeneic cell therapy with reduced risk of graft versus host disease (GvHD) toxicity or requirement for immunosuppressive agents.

Therefore, the TCB-202-001 trial investigated the safety and feasibility of infusion of *ex vivo* expanded γδ T cell products derived from partially HLA-matched family donors of patients with AML. One patient achieved an objective response categorized as complete response and one patient achieved MLFS. The contribution of lymphodepleting chemotherapy (Fludarabine) and direct alloreactivity due to HLA mismatch to AML cytoreduction in these patients is unknown and cannot be ruled out. Adverse events observed during the trial reflect the serious clinical condition of patients with active relapsed/refractory AML. No significant adverse reactions related to the study medication were seen. Significantly, there were no cases of DLT, infusion reactions, neurotoxicity or GvHD.

In the patient achieving MLFS, despite the MRD levels showing a sustained reduction from the baseline value, there was subsequent increase in bone marrow blast count after the second and third infusions indicative of disease progression. In studies with αβ T cells, leukemic blasts are known to adopt multiple mechanisms of immune evasion including secretion of immunoinhibitory factors inducing regulatory T cells (Tregs)^15^ and increasing the number of myeloid-derived suppressor cells (MDSCs)^16^ causing T-cell tolerance and disease progression. Overexpression of immune checkpoint ligands such as programmed cell death ligand 1 (PD-L1) has also been shown to drive T-cell exhaustion.^17^ However, few studies have focused on γδ T cells and the specific mechanisms involved in the context of immune tolerance and disease progression were not evaluated in this preliminary study.

A limitation of allogeneic γδ T cell approaches is the risk of graft rejection. In our study, lymphodepleting conditioning chemotherapy was used, both to limit rejection and to create space and access to homeostatic cytokines to promote expansion.^18^ One solution to graft rejection is repeat infusions to sustain responses. In our preliminary study, we have shown this was well tolerated in 3 patients without further preconditioning.

The promising, preliminary data of a favorable safety profile in late-stage AML patients paves the way for future clinical studies looking at early-treatment AML patients not responding to initial therapy. We are, therefore, now progressing a phase II pivotal clinical trial using an off-the-shelf, unrelated allogeneic donor source of γδ T-cells expanded *ex vivo* in patients with myeloid blood cancers associated with both high (relapsed/refractory AML), and low (MRD-positive) disease burden. The transition to a fully allogeneic product represents an opportunity to explore the therapeutic potential of γδ T-cells across multiple indications.

### Clinical Practice Points

• Adoptive transfer of γδ T lymphocytes from haploidentical donors in patients with refractory/relapsed acute myeloid leukemia was shown to be feasible and safe in a phase I trial.
  Further research on γδ T lymphocyte immunotherapy is warranted.

## Disclosure

TC BioPharm (TCB) received grants from Scottish Enterprise and the European Union during the conduct of the study. This project has received funding from the European Union's Horizon 2020 research and innovation program under grant agreement No 783506—OmnImmune. Jan Vydra has no disclosures. Emilio Cosimo is an employee of TCB. Petr Lesný has no disclosures. Richard Sebastian Wanless is an employee of TCB and also reports personal fees from Creative Monitoring Limited prior to becoming an employee. John Anderson holds founder stock in Autolus, share options in TCB and patents in CAR-T design. Alan George Clark is an employee of TCB and reports personal fees from Theraldia Consulting Limited during the conduct of the study. Angela Scott is an employee of TCB. Emma Kate Nicholson has received speakers’ fees, travel/conference grant and a research grant from KITE/Gilead, travel/conference grant from Novartis, travel/conference grant from Amgen and serves on advisory boards of Novartis, BMS/Celgene and Pfizer. Michael Leek is an employee of TCB.

## CRediT authorship contribution statement

**Jan Vydra:** Conceptualization, Investigation, Methodology, Project administration. **Emilio Cosimo:** Methodology, Investigation, Writing – review & editing. **Petr Lesný:** Investigation, Methodology. **Richard Sebastian Wanless:** Data curation, Formal analysis, Writing – review & editing. **John Anderson:** Conceptualization, Investigation, Writing – original draft. **Alan George Clark:** Methodology, Investigation, Writing – review & editing. **Angela Scott:** Conceptualization, Project administration, Supervision, Resources. **Emma Kate Nicholson:** Conceptualization. **Michael Leek:** Conceptualization, Supervision, Writing – review & editing.

## Appendix Supplementary materials

Image, application 1
