# Supplementary material for: A Phase I Trial of Allogeneic γδ T Lymphocytes From Haploidentical Donors in Patients With Refractory or Relapsed Acute Myeloid Leukemia
Source: Clin Lymphoma Myeloma Leuk. 2023 May;23(5):e232–9. doi: 10.1016/j.clml.2023.02.003 (PMC10139146; doi:10.1016/j.clml.2023.02.003)
Supplement: Supplementary file 1 [file mmc1.docx]

**Suppl. Table 1A: TCB-202-001 Patient Eligibility Criteria**

| **Inclusion criteria** |
| --- |
| - History of acute myeloid leukaemia (initially diagnosed by presence of 20% or more blast cells with myeloid or monocytic differentiation confirmed by flow cytometry in peripheral blood or bone marrow) |
| - - Relapsed or refractory AML |
| - - AML relapse after intensive chemotherapy OR |
| - - AML relapse after allogeneic HCT OR |
| - - AML progression on low intensity therapy (low dose cytarabine, 5-azacytidine or decitabine) OR |
| - - No response to at least 4 cycles of low intensity therapy OR |
| - - AML refractory to 2 cycles of induction chemotherapy |
|  |
| - Presence of > 5% of blasts in bone marrow or peripheral blood smear |
| - Patient not eligible or does not consent to high dose salvage chemotherapy and/or allogeneic HCT |
| - Considered suitable for lymphodepleting chemotherapy |
| - Age 18 to 70 years old |
| - Life expectancy of at least 3 months |
| - Karnofsky performance status ≥ 70% |
| - Available related HLA=haploidentical or HLA-matched donor |
| - Ability to be off systemic prednisone and other immunosuppressive drugs for at least 3 days prior to γδ cells product infusion. Maintenance replacement steroid therapy is allowed. |
| - Patient able to understand and sign written informed consent |
|  |
| **Exclusion criteria** |
| - Uncontrolled infections |
| - Renal insufficiency: creatinine > 180 µmol/L or on dialysis |
| - Heart failure: EF < 40% |
| - Respiratory insufficiency: oxygen therapy required at inclusion in the study |
| - Significant liver impairment; bilirubin > µmol/L, AST or ALT > 4 times normal upper limit |
| - Treatment with bisphosphonates (≤ 2 months before start) |
| - Active autoimmune disease or GvHD |
| - Pregnant or breastfeeding |
| - Patient of fertile age not using two-barrier method of birth control |
|  |

**Suppl. Table 1B: TCB-202-001 Donor Eligibility Criteria**

| **Inclusion Criteria** |
| --- |
| - Healthy subjects ≥ 18 years old |
| - HLA-haploidentical or HLA-matched donor for patient |
| - Suitable proliferative capacity of gamma delta (γδ) T lymphocytes in a cell culture assay |
| - Subject able to understand and sign written informed consent |
|  |
| **Exclusion criteria** |
| - Uncontrolled infection (clinically, blood tests and physical examination) |
| - Positive test results for HIV1/2, Hepatitis B/C, HTLV1/2 and syphilis |
| - Treatment with bisphosphonates (≤2 months before start) |
| - History of autoimmune disease |
| - History of malignancy |
| - Confirmed or suspected Transmissible Spongiform Encephalopathies (TSEs), a neurological disease of unknown aetiology or those who are blood relatives of persons with a familial Creutzfeldt-Jakob Disease (CJD) |

**Pregnant or breastfeeding**

**Suppl. Table 2: TCB-202-001 Donor and Patient HLA Status**

| **Patient number** | **HLA locus** | **Donor HLA** | | **Patient HLA** | |
| --- | --- | --- | --- | --- | --- |
| **5002** | **A** | 02:01 | 26:01 | 02:01 | 24:02 |
|  | **B** | 44:27 | 57:01 | 35:02 | 44:27 |
|  | **C** | 06:02 | 07:04 | 04:01 | 07:04 |
|  | **DRB1** | 07:01 | 16:01 | 11:04 | 16:01 |
|  | **DQB1** | 03:03 | 05:02 | 03:01 | 05:02 |
|  | **DPB1** | 04:01 |  | 04:01 |  |
| **5003** | **A** | 02:01 | 30:04 | 03:01 | 30:04 |
|  | **B** | 18:01 | 41:01 | 07:02 | 41:01 |
|  | **C** | 07:01 | 17:01 | 07:02 | 17:01 |
|  | **DRB1** | 03:01 | 11:04 | 03:01 | 15:01 |
|  | **DQB1** | 02:01 | 03:01 | 02:01 | 06:02 |
|  | **DPB1** | 02:01 | 104:01 | 02:01 | 104:01 |
| **5006** | **A** | 24:02 | 32:01 | 02:01 | 32:01 |
|  | **B** | 07:02 | 38:01 | 07:02 | 38:01 |
|  | **C** | 07:02 | 12:03 | 07:02 | 12:03 |
|  | **DRB1** | 04:04 | 13:03 | 01:01 | 13:03 |
|  | **DQB1** | 03:02 | 03:01 | 05:01 | 03:01 |
|  | **DPB1** | 04:01 | 06:01 | 02:01 | 04:01 |
| **5007** | **A** | 02 | 03 | 01:01 | 02:01 |
|  | **B** | 38 | 44 | 38:01 | 51:01 |
|  | **C** | 02 | 12 | 12:03 | 14:02 |
|  | **DRB1** | 01 | 13 | 13:01 |  |
|  | **DQB1** | 05 | 06 | not tested | |
|  | **DPB1** | 04:01 | 04:02 | 02:01 | 04:01 |
| **5008** | **A** | 02:01 | 03:01 | 03:01 | 32:01 |
|  | **B** | 07:02 | 44:02 | 07:02 | 40:02 |
|  | **C** | 03:04 | 07:02 | 02:02 | 07:02 |
|  | **DRB1** | 04:04 | 07:01 | 01:01 | 04:04 |
|  | **DQB1** | 03:02 | 02:02 | 05:01 | 03:02 |
|  | **DPB1** | 04:01 | 17:01 | 04:01 |  |
| **5009** | **A** | 25:01 | 32:01 | 24:02 | 32:01 |
|  | **B** | 18:01 | 57:01 | 18:01 | 48:01 |
|  | **C** | 06:02 | 07:01 | 07:01 | 08:03 |
|  | **DRB1** | 07:01 | 11:04 | 11:04 | 14:05 |
|  | **DQB1** | 03:03 | 03:01 | 03:01 | 05:03 |
|  | **DPB1** | 04:02 | 13:01 | 04:01 | 04:02 |
| **5010** | **A** | 02 | 24 | 01:01 | 02:01 |
|  | **B** | 18 | 27 | 18:01 | 49:01 |
|  | **C** | 02 | 07 | 07:01 |  |
|  | **DRB1** | 11 | 16 | 11:04 | 13:02 |
|  | **DQB1** | 03 | 05 | 03:01 | 06:04 |
|  | **DPB1** | 04 | 04 | 04:01 | 13:01 |
| **5011** | **A** | 01:01 | 02:01 | 02 | 03 |
|  | **B** | 44:02 | 50:01 | 44 | 51 |
|  | **C** | 05:01 | 06:02 | 05 | 15 |
|  | **DRB1** | 07:01 | 12:01/12:10 | 12 | 13 |
|  | **DQB1** | 02:02 | 03:01 | 03 | 06 |
|  | **DPB1** | 04:01 | 104:01 | 04:01 | 04:02 |

| **Suppl. Table 3A: TCB-202-001 Summary of TEAEs by System Organ Class, Term and CTC Grade** | | | | | |
| --- | --- | --- | --- | --- | --- |
|  | **CTC Grade 1** | **CTC Grade 2** | **CTC Grade 3** | **CTC Grade 4** | **CTC Grade 5** |
| **SOC PT** | n of Events (%) | n of Events (%) | n of Events (%) | n of Events (%) | n of Events (%) |
| **Infections and infestations** | **0** | **2 (28.6)** | **1 (14.3)** | **0** | **1 (14.3)** |
| Candida infection | 0 | 1 (14.3) | 0 | 0 | 0 |
| Clostridium difficile colitis | 0 | 0 | 1 (14.3) | 0 | 0 |
| Pneumonia | 0 | 0 | 0 | 0 | 1 (14.3) |
| Respiratory syncytial virus infection | 0 | 0 | 1 (14.3) | 0 | 0 |
| Rhinitis | 1 (14.3) | 0 | 0 | 0 | 0 |
| Sepsis | 0 | 1 (14.3) | 0 | 0 | 0 |
| Upper respiratory tract infection | 0 | 1 (14.3) | 0 | 0 | 0 |
| **Blood and lymphatic system disorders** | **0** | **0** | **0** | **3 (42.9)** | **0** |
| Thrombocytopenia | 0 | 1 (14.3) | 0 | 2 (28.6) | 0 |
| Neutropenia | 0 | 0 | 0 | 2 (28.6) | 0 |
| Anaemia | 0 | 1 (14.3) | 0 | 0 | 0 |
| Febrile neutropenia | 0 | 0 | 1 (14.3) | 0 | 0 |
| Leukopenia | 0 | 0 | 0 | 1 (14.3) | 0 |
| **Investigations** | **1 (14.3)** | **1 (14.3)** | **1 (14.3)** | **0** | **0** |
| Alanine aminotransferase increased | 0 | 1 (14.3) | 1 (14.3) | 0 | 0 |
| C-reactive protein increased | 0 | 2 (28.6) | 0 | 0 | 0 |
| Aspartate aminotransferase increased | 0 | 1 (14.3) | 0 | 0 | 0 |
| Blood bilirubin increased | 1 (14.3) | 0 | 0 | 0 | 0 |
| **Metabolism and nutrition disorders** | **1 (14.3)** | **1 (14.3)** | **1 (14.3)** | **0** | **0** |
| Hyperkalaemia | 2 (28.6) | 0 | 0 | 0 | 0 |
| Hypokalaemia | 0 | 1 (14.3) | 1 (14.3) | 0 | 0 |
| Hypomagnesaemia | 0 | 0 | 1 (14.3) | 0 | 0 |
| Hyponatraemia | 0 | 0 | 1 (14.3) | 0 | 0 |
| **Musculoskeletal and connective tissue disorders** | **1 (14.3)** | **2 (28.6)** | **0** | **0** | **0** |
| Back pain | 1 (14.3) | 0 | 0 | 0 | 0 |
| Bone pain | 0 | 1 (14.3) | 0 | 0 | 0 |
| Musculoskeletal chest pain | 0 | 1 (14.3) | 0 | 0 | 0 |
| Musculoskeletal pain | 0 | 1 (14.3) | 0 | 0 | 0 |
| **Skin and subcutaneous tissue disorders** | 1 (14.3) | 2 (28.6) | 0 | 0 | 0 |
| Rash | 1 (14.3) | 1 (14.3) | 0 | 0 | 0 |
| Erythema | 1 (14.3) | 0 | 0 | 0 | 0 |
| Skin ulcer | 1 (14.3) | 0 | 0 | 0 | 0 |
| Urticaria | 0 | 1 (14.3) | 0 | 0 | 0 |
| **Gastrointestinal disorders** | **1 (14.3)** | **0** | **0** | **1 (14.3)** | **0** |
| Diarrhoea | 1 (14.3) | 0 | 0 | 0 | 0 |
| Gastrointestinal haemorrhage | 0 | 0 | 0 | 1 (14.3) | 0 |
| Nausea | 0 | 1 (14.3) | 0 | 0 | 0 |
| Stomatitis | 0 | 1 (14.3) | 0 | 0 | 0 |
| Vomiting | 1 (14.3) | 0 | 0 | 0 | 0 |
| **General disorders and administration site conditions** | **2 (28.6)** | **0** | **0** | **0** | **0** |
| Fatigue | 2 (28.6) | 0 | 0 | 0 | 0 |
| Pyrexia | 1 (14.3) | 0 | 0 | 0 | 0 |
| **Cardiac disorders** | **0** | **1 (14.3)** | **0** | **0** | **0** |
| Atrial fibrillation | 0 | 1 (14.3) | 0 | 0 | 0 |
| **Eye disorders** | **1 (14.3)** | **0** | **0** | **0** | **0** |
| Conjunctival haemorrhage | 1 (14.3) | 0 | 0 | 0 | 0 |
| **Hepatobiliary disorders** | 0 | 0 | 1 (14.3) | 0 | 0 |
| Liver disorder | 0 | 0 | 1 (14.3) | 0 | 0 |
| **Immune system disorders** | 1 (14.3) | 0 | 0 | 0 | 0 |
| Cytokine release syndrome | 1 (14.3) | 0 | 0 | 0 | 0 |
| **Nervous system disorders** | 1 (14.3) | 0 | 0 | 0 | 0 |
| Ageusia | 1 (14.3) | 0 | 0 | 0 | 0 |
| Headache | 1 (14.3) | 0 | 0 | 0 | 0 |
| **Respiratory, thoracic and mediastinal disorders** | 0 | 1 (14.3) | 0 | 0 | 0 |
| Hypoxia | 0 | 1 (14.3) | 0 | 0 | 0 |
| **Vascular disorders** | 0 | 1 (14.3) | 0 | 0 | 0 |
| Hypotension | 0 | 1 (14.3) | 0 | 0 | 0 |
| **TEAE**: treatment emergent adverse event; **SOC**: system organ class; **PT**: preferred term | | | | | |

| **Suppl. Table 3B: Treatment Emergent Adverse Events by SOC and maximum reported CTCAE Grade** | | |
| --- | --- | --- |
| **SOC** | **Maximum reported CTCAE Grade** | **Number of events** |
| **Skin and subcutaneous tissue disorders** | **Total** | **8** |
|  | Grade 1 | 6 |
|  | Grade 2 | 2 |
| **Blood and Lymphatic Disorders** | **Total** | **13** |
|  | Grade 1 | 2 |
|  | Grade 2 | 2 |
|  | Grade 3 | 4 |
|  | Grade 4 | 5 |
| **Cardiac Disorders** | **Total** | **1** |
|  | Grade 2 | 1 |
| **Investigations** | **Total** | **13** |
|  | Grade 1 | 5 |
|  | Grade 2 | 7 |
|  | Grade 3 | 1 |
| **Gastrointestinal disorders** | **Total** | **7** |
|  | Grade 1 | 2 |
|  | Grade 2 | 3 |
|  | Grade 3 | 1 |
|  | Grade 4 | 1 |
| **Metabolism and nutrition disorders** | **Total** | **6** |
|  | Grade 1 | 2 |
|  | Grade 2 | 1 |
|  | Grade 3 | 3 |
| **Eye disorders** | **Total** | **3** |
|  | Grade 1 | 3 |
| **General disorders and administration site conditions** | **Total** | **7** |
|  | Grade 2 | 5 |
|  | Grade 4 | 1 |
|  | Grade 5 | 1 |
| **Vascular disorders** | **Total** | **1** |
|  | Grade 2 | 1 |
| **Nervous system disorders** | **Total** | **3** |
|  | Grade 1 | 3 |
| **Musculoskeletal and connective tissue disorders** | **Total** | **4** |
|  | Grade 1 | 1 |
|  | Grade 2 | 3 |
| **Immune System Disorders** | **Total** | **1** |
|  | Grade 1 | 1 |
| **Respiratory, thoracic and mediastinal disorders** | **Total** | **5** |
|  | Grade 1 | 1 |
|  | Grade 2 | 3 |
|  | Grade 3 | 1 |
| **NCI-CTCAE:** National Cancer Institute’s Common Criteria for Adverse Events, version 5, 27^th^. Nov. 2017  **TEAE:** treatment emergent adverse event  **SOC:** system organ class  **PT:** preferred term | | |

| **Suppl. Table 4: Potency of expanded Vγ9Vδ2 T cells.** | |
| --- | --- |
| **Manufacture Run** | **Potency** |
| TCB-202.PRA1-5002.01 | 71.46% |
| TCB-202.PRA1-5002.03 | 83.39% |
| TCB-202.PRA1-5003.01 | 62.11% |
| TCB-202.PRA1-5006.01 | 33.23% |
| TCB-202.PRA1-5006.02 | 41.53% |
| TCB-202.PRA1-5006.03 | 38.11% |
| TCB-202.PRA1-5007.01 | 17.71% |
| TCB-202.PRA1-5007.02 | 24.21% |
| TCB-202.PRA1-5007.03 | 30.60% |
| TCB-202.PRA1-5008.01 | 57.88% |
| TCB-202.PRA1-5009.01 | 27.52% |
| TCB-202.PRA1-5010.01 | 72.71% |
| To determine the potency of expanded Vγ9Vδ2 T cells a K562-based potency assay was performed for each manufacture run by the Quality Control department. Briefly, Vγ9Vδ2 T cells (E) and PKH67-labelled K-562 cells (T) were co-cultured at a E:T ratio of 10:1 at approximately 37 °C – 5% CO_2_ for 2.5 hours. The level of cell death of PKH67-labelled K-562 cells was then analysed by flow cytometry using Annexin-V (Biolegend, San Diego, CA, USA) and Propidium Iodide (Sigma-Aldrich, Gillingham, UK).  Potency (Specific cell death - %) was calculated with the following formula:  $\frac{\text{Sample Cell Death }\left( \text{\%} \right) \text{-}\text{ }\text{Spontaneous Cell Death }\left( \text{\%} \right)}{\text{100}\text{ }\text{-}\text{ }\text{Spontaneous Cell Death }\left( \text{\%} \right)\text{ }}\text{x 100}$ | |
